# Takotsubo Syndrome: The Secret Crosstalk between Heart and Brain

**DOI:** 10.31083/j.rcm2401019

**Published:** 2023-01-10

**Authors:** Sofia Schino, Andrea Bezzeccheri, Alessandro Russo, Michela Bonanni, Joseph Cosma, Giuseppe Sangiorgi, Gaetano Chiricolo, Eugenio Martuscelli, Francesco Santoro, Enrica Giuliana Mariano

**Affiliations:** ^1^Department of Cardiovascular Medicine, University of Rome “Tor Vergata'', 00133 Rome, Italy; ^2^Department of Biomedicine and Prevention, University of Rome “Tor Vergata'', 00133 Rome, Italy; ^3^Department of Medical and Surgery Sciences, University of Foggia, 71122 Foggia, Italy

**Keywords:** Takotsubo, cardiomyopathy, broken heart syndrome, stress-induced cardiomyopathy

## Abstract

An acute, transient episode of left ventricular dysfunction characterizes Takotsubo syndrome. It represents about 2% of all cases of acute coronary syndrome (ACS), and occurs predominantly in postmenopausal women, generally following a significant physical or emotional stressor. It can be diagnosed based on clinical symptoms and the absence of coronary artery disease on angiography. Ventriculography remains the gold standard for the diagnosis. Despite its transitory characteristic Takotsubo syndrome should not be considered a benign condition since complications occur in almost half of the patients, and the mortality rate reaches 4–5%. Lately, it has been revealed that Takotsubo syndrome can also lead to permanent myocardial damage due to the massive release of catecholamines that leads to myocardial dysfunction. Different mechanisms have been advanced to explain this fascinating syndrome, such as plaque rupture and thrombosis, coronary spasm, microcirculatory dysfunction, catecholamine toxicity, and activation of myocardial survival pathways. Here are still several issues with Takotsubo syndrome that need to be investigated: the complex relationship between the heart and the brain, the risk of permanent myocardial damage, and the impairment of cardiomyocyte. Our review aims to elucidate the pathophysiology and the mechanisms underlying this complex disease to manage the diagnostic and therapeutic algorithms to create a functional synergy between physicians and patients.

## 1. Introduction 

Takotsubo syndrome (TTS) is an acute type of heart failure characterized by reversible left ventricular dysfunction, also known as stress cardiomyopathy, “broken heart syndrome” [1, 2], or “happy heart syndrome” [3]. It was first described in 1983 [4] at the Hiroshima City Hospital in a 64-year-old female complaining of chest pain with ST-segment elevation on the electrocardiogram (ECG) heart in the absence of coronary artery lesions on angiography. The ventriculography showed an “octopus trap” shaped ventricle (the so-called “takotsubo,” as defined by Sato *et al*. in 1990 [5]). Initially considered rare, benign, and self-limiting, TTS is now considered a syndrome with increased morbidity and mortality and can recur.

## 2. Epidemiology 

TTS represents almost 2% of cases of acute coronary syndrome [6]. In 90% of cases, it occurs in postmenopausal women [7], and the outbreak is usually linked to a significant physical or emotional stressor. The average age is 60–70 years, but it usually affects people over 50 years [8]. Nevertheless, TTS must not be considered a gender-related pathology. Among increasing awareness of TTS, numerous cases have been found in male patients (10% of cases), usually secondary to a physical stressor [9]. The literature reports some rare instances of TTS affecting children (the youngest being a premature baby in the 28th gestational week) [10]. There are no data about possible interracial differences, but it seems more common among Caucasians and less among Hispanics and African-Americans, despite the latter having more in-hospital complications and some recurrent ECG changes such as QT corrected (QTc) elongation and T-wave inversions [11].

## 3. Etiology 

TTS was previously known as the “broken heart syndrome” [1, 2] since it was frequently related to negative stressors. The crucial role of a sudden massive release of catecholamines in the physiopathology of this syndrome was revealed in 2005 by Wittstein *et al*. [12]. TTS has been linked to sympathetic hyperactivation during an extreme stress event (both physical and emotional). Some environmental triggers could also cause TTS (i.e., deafening noise). TTS can occur spontaneously, with no underlying stressor. Takotsubo syndrome may also be caused by medical treatment (so-called iatrogenic), such as administration of Sympatico-mimetic drugs while performing tests (i.e., dobutamine in stress-echo procedures or isoproterenol in intracardiac electrophysiology studies), or by beta agonists drugs [13]. Other clinical conditions have been linked to this syndrome, such as pheochromocytoma [14], acute respiratory insufficiency, sepsis [15], stroke [16], and migraines [17]. Acute myocardial infarction (MI) must be excluded when treating a patient with acute ST-segment elevation. MI could also be the trigger that causes TTS, secondary to the intense stress caused by the event.

## 4. Pathophysiology of TTS 

**Fig. 1.**
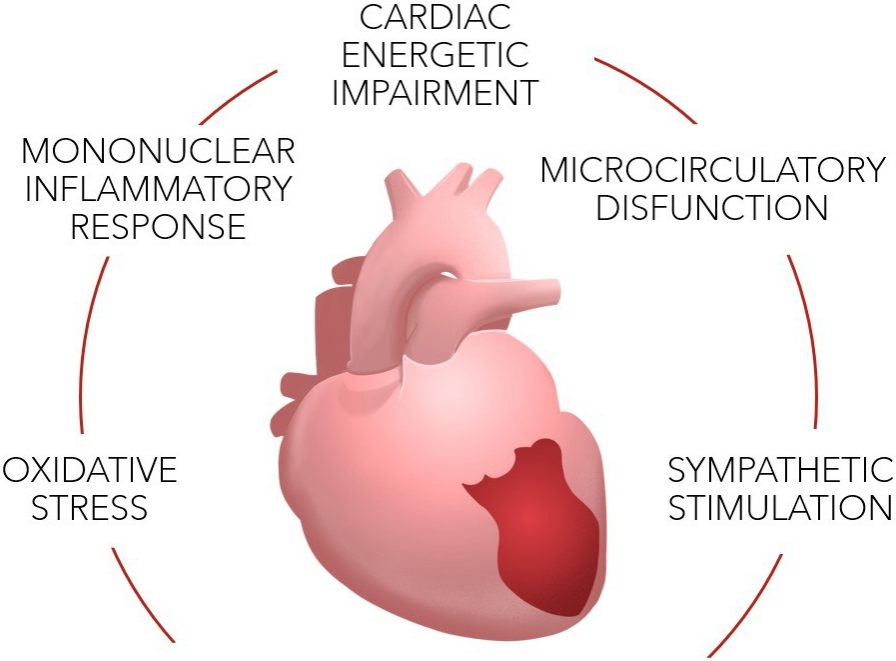
**Pathophysiology of TTS**.

The term “syndrome” comes from the Greek “συνδρoμή” which means “running together”: this stands for the combination of both signs and symptoms that can be related to this pathology (See Fig. 1) [2, 6, 9].

**Fig. 2.**
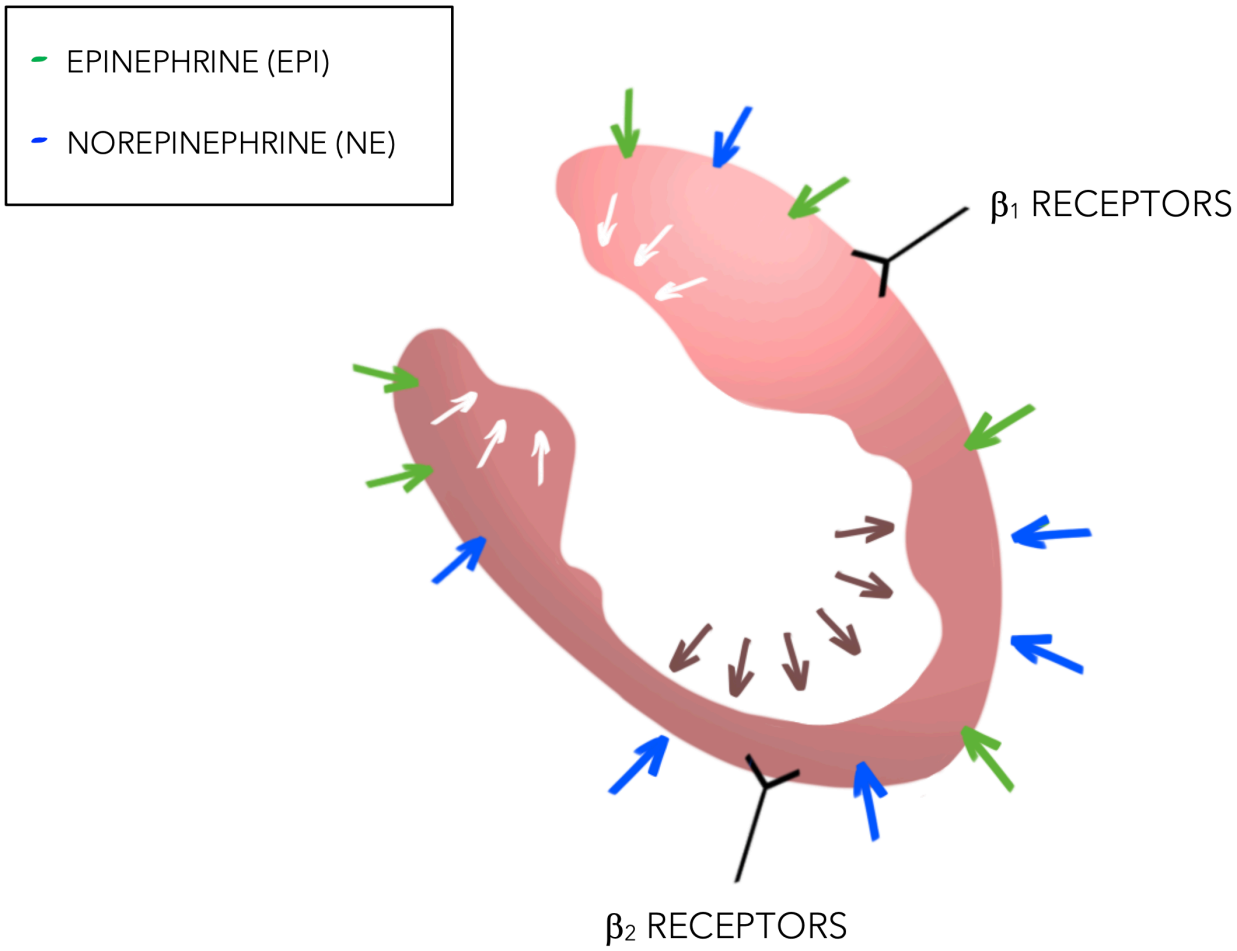
**Sympathetic stimulation of the heart in TTS**.

**Sympathetic stimulation**. As previously noted, sympathetic stimulation plays a key role in the correlation between the presence of a stressor and a precipitating factor. Other studies on heart variability rate (HVR) have shown high sympathetic activity with marked depression of the parasympathetic pathway [18]. A study focusing on analyzing biopsies on six human hearts [19] established that both adrenergic and cholinergic fibers cross the myocardial tissue from the epicardial layer to the myocardial layer, along with coronary vessels. In the subepicardial layer there is a higher number of adrenergic terminations, while cholinergic ones are prevalent in the subendocardial layer. Moreover, there is a difference in distribution of the innervation; the anterior wall of the left ventricle is richer than the posterior wall. In addition, the left ventricle has a lower density of cholinergic fibers than the right ventricle. Consequently, the trigger which may cause TTS provokes a hyperactivation of the sympathetic fibers normally counterbalanced by the parasympathetic system. Norepinephrine may cause a stimulating effect on β1 receptors resulting in an increased contractility at the base of the left ventricle (Lyon *et al*. [20]). On the other hand, epinephrine stimulates β2 receptors, which provokes a Gs to Gi switch that induces a negative inotropic effect at the apex. This theory may explain the apoptotic changes that have been shown at the apex of the left ventricle, secondary to the excessive stimulation of β2 receptors [20]. The contrast between the apical stunning (due to the stimulation of β2 receptors) and the hypercontractility of the base (β1) increases the end systolic pressure of the LV which creates the apical ballooning effect (See Fig. 2). In this setting, it has been hypothesized that this negative inotrope response may be a protective mechanism which limits myocardial damage. This may explain why an improvement of the EF is often seen after hours, weeks or months [21].

It’s interesting to note that only some of these secreted catecholamines reach the bloodstream; the majority are sent to the myocardial adrenoreceptors directly by the sympathetic nerve ends [22]. In particular, during the acute phase of TTS there could be a greater myocardial catecholamine release rather than in the adrenal gland [23].

High concentrations of catecholamines can have toxic effects on cardiomyocytes causing a mononuclear inflammatory response. Necrosis transverse bands as shown in endomyocardial biopsies, have been found in TTS patients [24]. The excess catecholamines could induce oxidative stress and mediate an inflammatory reaction with the release of interleukin [11]. This mechanism may be also related to activation of myocardial survival pathways with direct inhibition of apoptotic mechanisms and inhibition of cellular function in order to preserve cell functionality in a condition characterized by myocardial stunning [25].

Elevated norepinephrine levels also correlate with increased C-reactive protein levels and leukocyte counts in acute TTS, suggesting that catecholamines may cause a more systemic inflammatory response [26]. In addition, it has been demonstrated (Nef *et al*. [27]) that individuals suffering from TTS had a significant macrophage infiltration within the myocardium. Macrophages contain phagocytic NADPH oxidase (NOX-2) which is vital in controlling oxidative stress in the myocardium and vascular (endothelial) cells.

Although it is evident that these factors have a fundamental role in TTS, they unlikely to explain all the causes TTS, since every person experiencing a stressful situation would suffer from TTS. It has been proposed that high levels of catecholamines may be the fundamental mechanism for this syndrome [28].

**Plaque rupture and thrombosis**. It has been suggested that TTS may be the consequence of plaque rupture, thrombosis and rapid lysis of the thrombus. This hypothesis comes from the frequent (18–29%) findings of atherosclerotic plaques in patients suffering from TTS [29]. Although some studies have been conducted using intravascular imaging, ulcerated plaques and thrombi have been uncommon findings [30, 31]. In addition, myocardial necrosis biomarkers are decreased in TTS compared to an MI, and the wall motion abnormalities usually extend beyond single coronary artery territories [4].

**Multi-vessel epicardial spasm**. Data from the literature confirm that the endothelium may play a fundamental role in catecholamine release and that endothelial dysfunction may be present in patients affected by TTS. This hypothesis is linked to the anamnestic match between TTS and both migraines and Raynaud’s phenomenon [7]. TTS patients have also marked impairment in brachial-artery flow-mediated dilatation, which tends to improve with time [12]. Some but not all patients suffering from TTS have a predisposition to vascular spasms, demonstrated by injection of acetylcholine in the epicardial vessels [32]. Moreover, high levels of circulating catecholamines reduce receptivity to vasodilatory mediators. This may be the cause of a sudden drop in flow-mediated vasodilatation at admission, whereas a gradual drop in catecholamine levels in the blood may result in an increase in flow-mediated vasodilatation values at discharge [33]. Furthermore, different grades of perfusion (TIMI) have been observed in angiographic studies [34]. These findings suggest that the flow and the perfusion in the LAD territory are impaired regardless of the morphological pattern. In 2018 a retrospective study was performed using quantitative evaluation of coronary flow, measured with TIMI frame count (TFC) in patient affected by TTS [35]. TFC is the number of frames the contrast requires to reach distal landmarks [36]. TFC is augmented in the LAD of patients suffering from TTS, and confirms that the impaired flow in the coronary arteries are probably due to vascular dysfunction.

**Microcirculatory dysfunction and oxidative stress**. It has been also shown that intravenous administration of adenosine provokes a transient improvement of myocardial perfusion and of contractility index, suggesting that an intense microvascular constriction might play a crucial role in the pathogenesis of TTS [37]. Adenosine-mediated vasodilatation in smooth muscle and vascular cells counterbalances the intense catecholamine-related vasoconstriction, probably due to the activation of Gs protein [37]. On the basis of the pathognomonic apical-ballooning pattern, it has been proposed that some alterations may implicate the territory of the left descendent artery (LAD) [37], but this hypothesis has not been validated.

Microvascular dysfunction can be assessed by using the index of microvascular resistance (IMR) or as an alternative Coronary flow reserve (CFR). In 2011 Kim *et al*. [38] evaluated 11 patients with TTS with 12 patients with an anterior STEMI. They found evidence that the microcirculatory function is markedly altered in TTS but it is more reversible than that detected in patients after a STEMI. In 2017 [39] a study based on a prospective cohort of consecutive patients with TTS found that the myocardial damage, demonstrated by IMR, resolves in a time-dependent manner, and maybe responsible for the recovery seen with myocardial stunning.

In addition, endothelial dysfunction and underlying oxidative stress, may also be important mechanisms in TTS. In 2015 Nanno *et al*. [40] studied 8- hydroxy-2’-deoxyguanosine (8-OHdG) as a potential biomarker of oxidative stress, secondary to the effects of reactive oxygen species (ROS) on DNA. These authors measured 8-OHdG and norepinephrine levels in patients with a MI and TTS and showed that patients with TTS have values twice as high as in MI. In 2020 a study by Mao *et al*. [41] documented the overexpression of phosphoinositide 3-kinase/protein kinase B/mammalian target of rapamycin (PI3K/AKT/mTOR) pathways in TTS induced in rats, consequently inducing apoptosis and oxidative stress. In contrast, the same data showed that chronic inhibition of the PI3K/AKT/mTOR pathway may have a protective role, reducing mitochondrial ROS and oxidative stress-induced apoptosis [41].

Myocardial cells may be directly injured by increased ROS production and increased catecholamine release in TTS. Furthermore, it has been demonstrated that direct exposure of cardiac cells to ROS results in a loss of systolic contractility, resulting in diastolic dysfunction, metabolic malfunction, and depletion of high energy phosphates [42].

It has been proposed that left ventricular ballooning may be more likely to occur in patients due to changes in erythrocyte membranes and endothelial integrity brought on by catecholaminergic storm [43].

Recent evidence suggests that increased nitrogen monoxide (NO) sensitivity may play a significant role in the pathophysiology of TTS. Nguyen *et al*. [44] found that platelets from 56 TTS patients had significantly higher NO responsiveness, which may substantially impact the severity of the initial myocardial injury. Translational studies in rats show the importance of nitrosative stress and activation of poly (ADP ribose) polymerase-1 (PARP-1) [44]. Notably, pretreatment with a PARP-1 inhibitor decreased nitro-oxidative/nitrosative stress and attenuated negative inotropic changes, indicating that the peroxynitrite/PARP-1 cascade may be responsible for the negative inotropy in this model of TTS.

Other biomarkers of endothelium damage have also been investigated during the acute phase of TTS and at follow-up. The findings of this investigation support the theory that endothelial dysfunction and hyperviscosity play a role in the pathogenesis of TTS. Additionally, they imply that changes in erythrocyte deformability and endothelial dysfunction continue beyond the acute period and may be the focus of therapeutic techniques used to treat TTS [45].

**Fig. 3.**
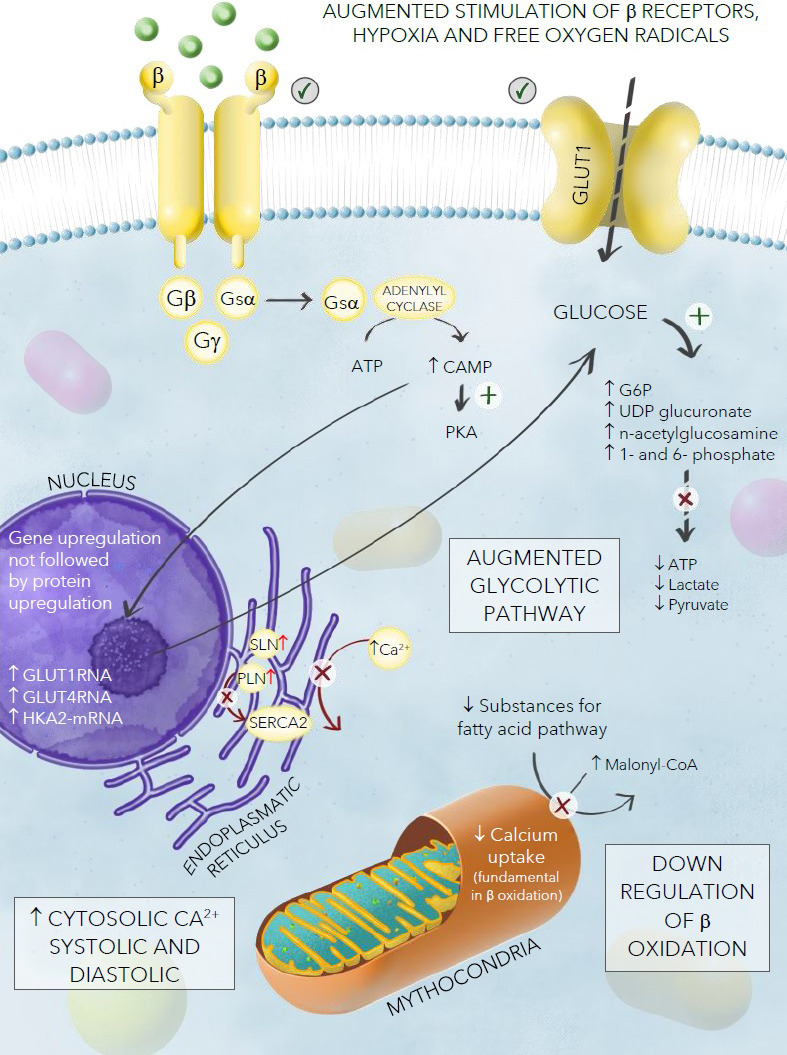
**Model of energetic cardiac impairment in TTS**.

**Cardiac energetic impairment **(See Fig. 3).

**Different metabolic pathways in TTS**. Recently, Godsman *et al*. [46] analyzed metabolic alterations in isoprenaline-induced TTS in a rat model. This impaired energetic status appears to be related to an augmented glycolytic pattern, and is confirmed by high levels of GLUT4-RNA, hexokinase 2 (HKA2)-mRNA, and increased metabolites such as glucose-6-phosphate (G6P), uridine diphosphate glucuronic acid (UDP) glucuronate, n-acetylglucosamine 1-phosphate, and n-acetylglucosamine 6-phosphate. End stage metabolites such as lactate and pyruvate (along with the generation of ATP) were decreased. This could be explained by noting that the adrenergic stimulus increases metabolism in cardiac cells, consuming the final Krebs cycle intermediates. In addition, the GLUT1 membrane transporter, involved in myocardial stress responses and expressed on the surface of infiltrating macrophages, was augmented in the apex of this TTS model. The authors also found downregulation of beta-oxidation, confirmed by the augmentation of Malonyl-CoA, which regulates the ingress and oxidation of fatty acids in mitochondria, and the reduction of both cytosolic substrates for the fatty acid pathway (except for palmitate, which may remain protected) and mitochondrial metabolites. All these elements support a cross-regulation between these two pathways (glycolysis and beta-oxidation), which seems to be fundamental in TTS.

**The role of calcium ions**. Calcium ions play a crucial role in the cardiac muscle fibers, regulating excitation-contraction and relaxation. A significant alteration in calcium flux results in high levels of both systolic and diastolic calcium concentration but decreased mitochondrial calcium uptake with a subsequent decrease of these ions in the mitochondria; which is fundamental in the process of beta-oxidation. This may further worsen this energetic mismatch. The augmented stimulation of adrenoceptors, which enhances heart rate and cardiac contractility, causes an imbalance in the ratio of oxygen supply to oxygen demand, creates areas of myocellular hypoxia and changes in membrane permeability, causing altered cationic homeostasis which contributes to myocardial toxicity affecting cellular processes [47]. The presence of oxygen-derived free radicals released in response to norepinephrine and epinephrine may also interfere with calcium and sodium transporters, resulting in additional myocyte dysfunction [48]. The increase of catecholamine, which occurs in TTS, may cause hyperactivation of β1 and β2 beta-adrenergic receptors by activating adenylate cyclase by interacting with the stimulatory G protein. This leads to an intracellular increase of cyclic adenosine monophosphate that activates protein kinase A (PKA). The high intracellular calcium levels interfere with ventricular contraction and function, decreasing the viability of cardiac myocytes [8]. Moreover, in the sarcoplasmic reticulum (SR), factors such as the homologous intrinsic membrane proteins Sarcolipin (SLN) and Phospholamban (PLN), which are co-located in SR and are considered to be critical regulators of cardiac contractility, may also be affected.

It has been observed that patients affected by TTS have an unusually elevated ventricular expression of SLN that may play a crucial role in the calcium regulation process mediated by sarcoplasmic/endoplasmic reticulum calcium ATPase 2 (SERCA2) that lowers its affinity for calcium [49]. This calcium overload cAMP-mediated process results in contraction band necrosis which is one of the pathological hallmarks of TTS [50]. These elements are characterized by hypercontracted sarcomeres and dense eosinophilic transverse bands. In contrast with the polymorphonuclear inflammation seen in infarction, contraction band necrosis causes an interstitial mononuclear inflammatory response [50].

Finally, it is thought that a switch from aerobic to anaerobic metabolism is required to satisfy the abnormal increase in contractility due to the massive release of catecholamines [51].

**MiRNAs and TTS**. Micro-RNA (miRNA) which are upregulated by stressful situations in mice [52]; and may be important in diagnosing TTS. MicroRNAs (miRs) are small non-coding mRNA as long as 1000 nucleotides derived from more extended regions of RNA [53]. MiRs regulate cellular processes such as proliferation, differentiation, development, and cell death, acting as intracellular regulators of post-translational expression. MiRs have a tissue- and cell-specific expression profile: MiRs in systemic circulation may reflect tissue damage [54]. In 2013, Jaguszewski [55] found that miR1, miR16, miR26a, and miR133a are biomarkers of acute Takotsubo syndrome (TTS). MiR125a-5p down-regulation increases endothelin-1 plasma levels and may be implicated in the microvascular spasm physiopathology in TTS [55]. Bcl2-associated athanogene 3 (*BAG3*) is part of a family of co-chaperones interacting with the ATPase domain of heat shock protein 70 (*hsp70*). BAG3 expression is restricted to a few cell types, including cardiomyocytes [51, 56, 57]. Its expression can be induced by various stressors and contributes to stress resistance. There is some evidence that *BAG3* loss-of-function mutation is involved in cardiomyopathy because of alterations of myofibrillar integrity [58, 59]. TTS patients had a higher frequency of mutation of the *BAG3* gene than healthy donors. Exposure to epinephrine, either as a consequence of emotional stress or administration of catecholamines or the presence of an inhibitor of serotonin/norepinephrine re-uptake, has been implicated in TTS [60]. Treatment with epinephrine resulted in higher expression of miR371a, indicating that this miRNA may have a role in *BAG3* induction.

**Fig. 4.**
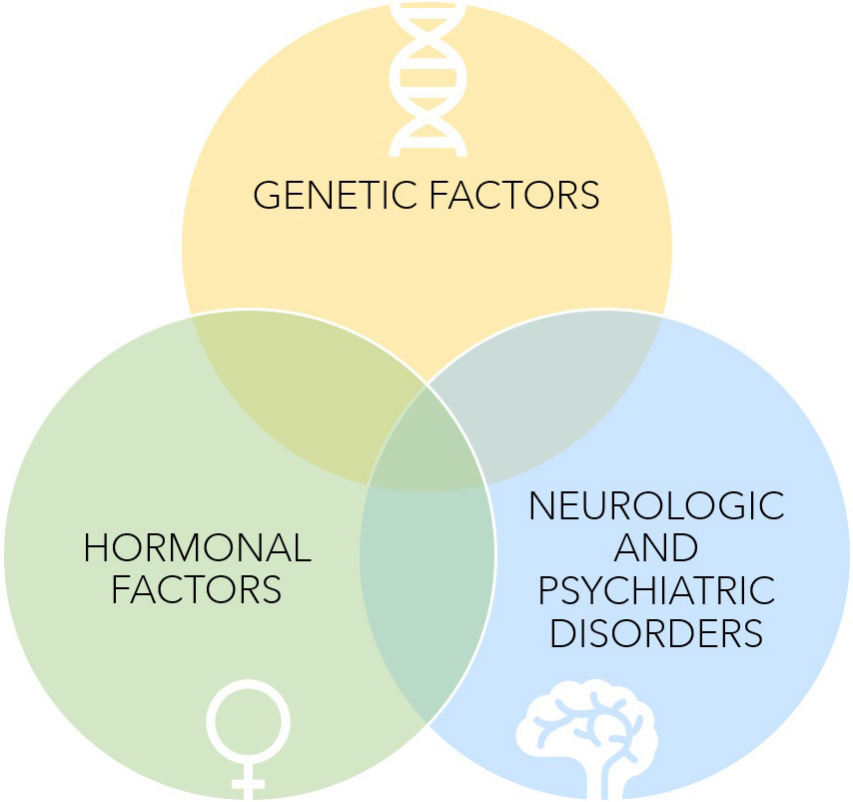
**Predisposition to Takotsubo Syndrome**.

**Predisposition and risks factors **(See Fig. 4).

Several risk factors have been proposed in establishing a predisposition for TTS. This syndrome is more frequent among post-menopausal women [7]. This is thought to be linked to the upregulation of estrogens on endothelial NO production and concomitant downregulation of sympathetic stimulation [31]. The use of estrogens in postmenopausal women seems to be associated with reduced vasoconstriction due to reduced stress-related sympathetic response [61, 62, 63]. Unfortunately, recent small retrospective studies have shown that the administration of hormones is not protective against TTS [64]. This suggests that while estrogens may have an additional role, it is not causative in TTS.

A second assumption is based on the presence of genetic factors supported by several cases of TTS within the same families reported in the literature [65]. However, there is no recognized mendelian transmission. Several studies have focused on different gene expressions (for instance, those regulating the expression of beta-adrenergic receptors), but none have been conclusive [66, 67, 68]. In the largest genetic study on TTS, 68 loci have been studied; unfortunately, none have shown sufficient statistical significance [69].

## 5. Diagnosis

**Fig. 5.**
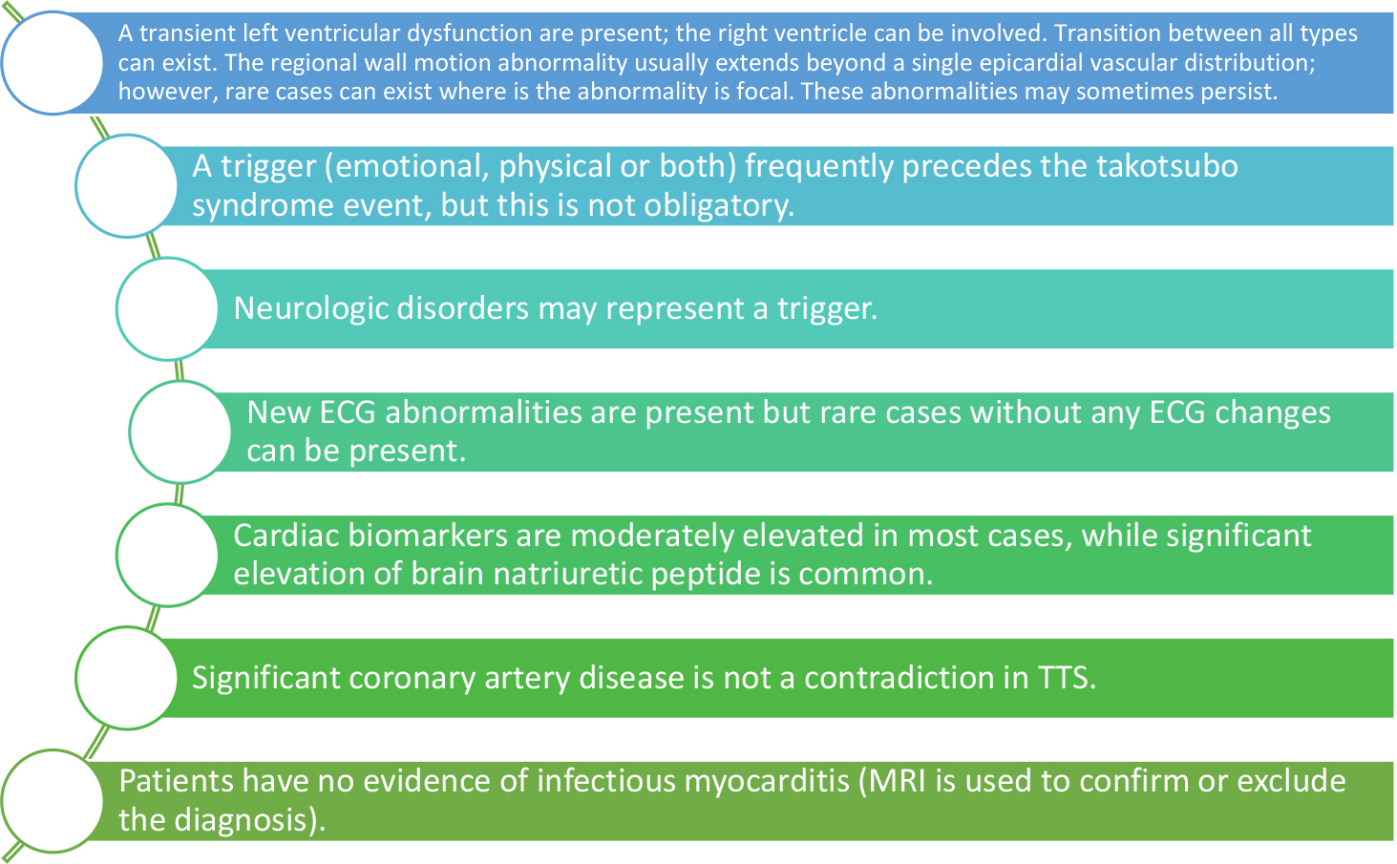
**InterTAK diagnostic criteria 2018**.

**InterTAK diagnostic criteria**. The diagnosis of TTS is complex and not widely accepted; different diagnostic criteria have been established in recent years. The revised Mayo Clinic Criteria and the International Takotsubo (InterTAK) Campo [70] diagnostic criteria are the most used scores. The InterTAK diagnostic criteria help determine the probability of having TTS. A value above 70 points stands for high probability and below 70 means low probability (See Fig. 5).

**Fig. 6.**
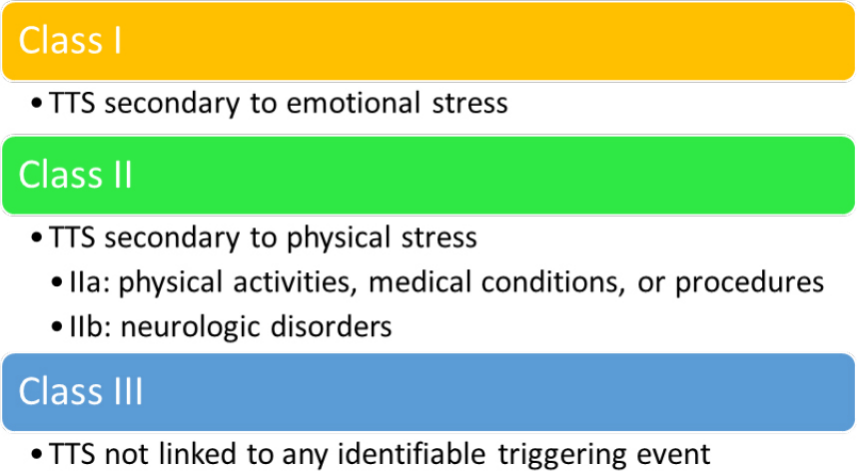
**2018 Classification of TTS (type of triggering event)**.

In 2018, a new classification based on the trigger event was proposed by Ghadri *et al*. [71] (See Fig. 6).

**Symptoms**. The most common symptoms are constrictive chest pain, dyspnea, and syncope [4]. Sometimes, there is a severe clinical presentation with cardiogenic shock, which can occur in 10% of cases [8], or major arrhythmias [4]. The presence of a stressful event in the five days [28] before the event may be helpful, but it is not always clear.

**ECG characteristics**. ECG changes are not a characteristic feature; and may resemble ACS (4). It has been observed that ST-segment depression in lead aVR was 95% specific for Takotsubo syndrome versus an MI [72]. It can show diffuse ST-segment elevation, more frequently in the anterior leads, inverted T waves, or ST depression (less than 10% of cases) [8]. Q waves, left bundle branch block, QRS fragmentation, and low voltages are far less common [73]. Similar abnormalities in the inferior leads may also be found. It may also show inverted T waves (in the same territories which presented with ST-segment elevation, probably an expression of myocardial stunning) [73] and QT corrected (QTc) elongation [74]. The latter constitutes a proarrhythmic substrate. It was observed that QTc intervals are associated with adverse cardiovascular outcomes in healthy and high-risk populations [75]. Prolonged QTc interval at admission seems to be associated with a higher risk of cardiovascular rehospitalization.

In contrast, a better prognosis is characterized by the dynamic increase of QTc intervals after admission [75]. It has been proposed that some ECG characteristics can suggest a worse prognosis, such as a triangular QRS ST-T waveform (the so-called “shark-fin” pattern) or persistent ST-segment elevation beyond 48 h from admission. It has been suggested that prolonged microvascular dysfunction may be responsible for these electrocardiographic changes [76]. The abnormalities seen in the ECG are usually temporary and normalize with time.

**Biomarkers**. Elevation of biomarkers of myocardial necrosis are commonly lower than expected compared to acute coronary syndromes [7]. It is believed to be secondary to the increase abundance of catecholamines rather than to necrosis of myocardial cells; the damage may be related to edema rather than to cellular death [77]. Higher levels of N-terminal prohormone of brain natriuretic peptide (NT-proBNP) are found in TTS compared to ACS; which peak in 24–48 h [78] and resolves within months [79]. Levels of NT-proBNP/troponin seems to have a tremendous discriminating power between TTS and ACS, as well as High-Sensitivity Troponin I (hs-TnI) and creatine kinase-myoglobin binding (CK-MB) rate. hs-TnI/CK-MB is also significantly higher in patients with TTS [79]. BNP elevation seems to be related to sympathetic hyperactivation, and reactive C protein (RCP), suggesting an inflammatory component, related to the presence of edema on magnetic resonance imaging (MRI) [55]. Serum levels of anti-inflammatory Interleukins 2, 4, and 10 are higher when compared to ACS, but their role for diagnostic purposes is still being studied [80].

**Angiography and ventriculography**. Angiography is fundamental to evaluate coronary arteries. In most cases, there is no narrowing, suggesting that they are not responsible for the symptomatology and laboratory findings. ACS and TTS may coexist in 18–29% [29] patients; for this reason, coronary anatomy should be carefully assessed using different projections. Ventriculography remains the gold standard for the diagnosis of TTS since it opacifies the left ventricle and can show five different patterns of ventricular abnormalities: the most common is the “apical ballooning” pattern, with hypercontractility of the basal segments and akinesia of the apex.

In about 30% of cases, the “apical nipple” sign can be present, where the apex of the heart has normal contractility; and can be useful to distinguish a patient suffering from TTS from one presenting with an AMI [81]. The second pattern is the “mid-ventricular” one, showing ipo-akinesia of the mid-ventricular segments and hypercontractility of the basal and apical ones, whose image on ventriculography resembles the “the hawk’s beak appearance” [82]. When the opacification of the left ventricle shows an ipo-akinesia of the basal segments and hypercontractility of the apex, it is called “basal (or reversed)”. It can also present with just one akinetic segment (usually anterolateral), resulting in a “focal” TTS pattern. There can also be an atypical form, with biventricular involvement or isolated akinesia of the right ventricle. These atypical forms seem to be more common in younger patients with neurological comorbidities, ST-segment depression, and lower levels of NT-proBNP [4]. Some anatomical variants (like myocardial bridging or tortuous arteries) are common, although not specific to TTS [19].

**Echocardiography**. Echocardiography is the most useful, rapid, and accessible tool to evaluate a patient complaining of chest discomfort. The different patterns are the same as described above. The wall abnormalities exceed the distribution of a single coronary artery. Areas affected by reduced myocardial function are typically opposite; this is called a “circumferential pattern” and represents a hallmark in diagnosing TTS [83]. Another vital marker of LV dysfunction is the E/e’ ratio, which usually improves with time [84]. Right ventricular (RV) assessment is also crucial [85]. Echocardiography is also helpful in detecting complications such as obstruction of the left ventricular outflow tract (LVOT), significant mitral insufficiency, presence of thrombi in the apex, and mechanical complications (like left ventricular wall rupture, for which prompt detection is fundamental for rapid referral for surgery) or pericardial effusions. LVOT obstruction (LVOTO) seems to be related to the pre-existent sigmoid septum, which is frequently seen in older age and post-menopausal women [86], small ventricular cavity, restriction of the outflow tract, and redundant mitral leaflets [87]. The mitral insufficiency seems to be a consequence of different mechanisms, among which tethering due to dislocation and dysfunction of the papillary muscle is the most common [88], but may also be related the presence of systolic anterior movement (SAM) related to LVOTO [89].

New techniques such as strain, strain rate, and speckle tracking will help to find other parameters in evaluating TTS. These techniques have shown that both systole and diastole are affected for long periods of time, extending far beyond the acute phase despite the recovery of EF [90]. The EF depression usually recovers in 4–8 weeks [8] along with LVOT obstruction. Therefore, it is recommended that daily assessment of myocardial function with echocardiography should be done in the acute phase.

**MRI**. An MRI is usually needed as it provides a more accurate evaluation of left ventricular function and possible complications such as pleural and pericardial effusions, and apical thrombi. Late gadolinium enhancement (LGE) imaging, helps to rule out the diagnosis of myocarditis [91]. The absence of a signal in the LGE sequences is characteristic of TTS since there is usually no fibrosis [92]. Unfortunately, some cases of TTS with hyperintensity on LGE sequences have been described in the literature, often associated with definitive and irreversible loss of contractility in the affected areas [93]. A recent prospective study has proposed the combination of T1 and T2 mapping sequences to identify acute myocardial injury without the need to use gadolinium contrast with reasonable accuracy [94]. Another peculiar feature of TTS is the presence of hyperintensity in STIR sequences, which confirms the presence of edema. Unfortunately, the mechanism responsible for developing myocardial edema remains unknown. Edema usually shows a transmural distribution, typically detectable in the acute phase, resolving along with LV recovery [95].

**Positron Emission Tomography (PET)**. PET helps study perfusion and metabolism using different traces. Perfusion scans do not usually show any alteration, which differs from metabolic ones using 18-fluorodesossiglucosium in which the trace is not picked up by cells in dysfunctional areas in TTS. The mechanisms underlying this effect are not well established. However, it is probably related to the massive release of catecholamines and the increased expression of beta receptor’s higher expression in the left ventricle’s apex [96]. Recent studies have shown that patients with TTS present with metabolic abnormalities in the acute and subacute phases of TTS, along with slightly reduced myocardial perfusion [97]. These metabolic alterations persist longer than LV contraction abnormalities, allowing TTS to be detected even in misdiagnosed patients.

**Single-photon Emission Computed Tomography (SPECT) with 123-Meta-iodobenzyl guanidine (123-MIBG)**. The role of nuclear medicine in diagnosing TTS is not well established. The use of some traces are fundamental to the understanding of the pathophysiology and mechanisms of TTS. The use of combined perfusion and metabolic images can help in distinguishing an AMI from TTS. 123I meta-iodobenzyl guanidine (MIBG) evaluates the cardiac adrenergic innervation, which is reduced in the regions affected by TTS, and differs from perfusion which remains normal [98]. In the subacute phase, the 123I-MIBG uptake is impaired, and lasts for a long time due to alterations in sympathetic activity [99].

**Fig. 7.**
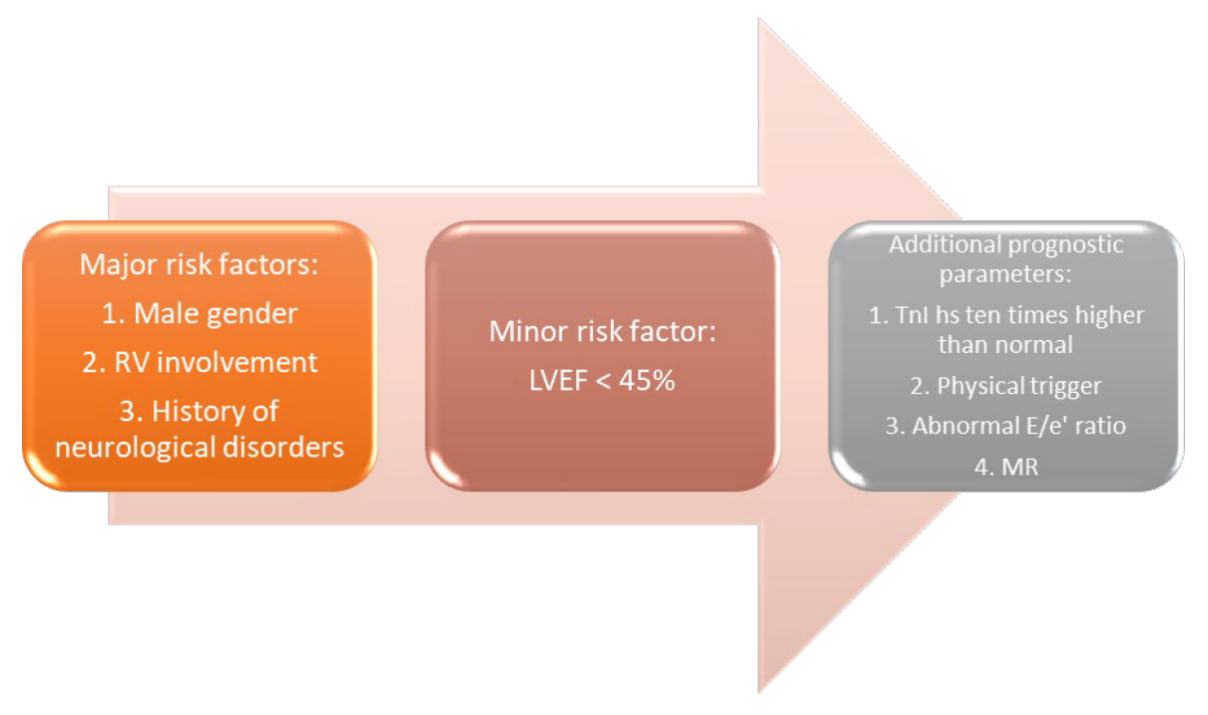
**In-hospital risk score developed from the German and Italian stress cardiomyopathy registry**.

**Complications and recurrence**. Cardiovascular complications can occur in almost 50% of TTS, and the mortality rate reaches 4–5% [4]. Some of the most serious complications include cardiogenic shock, ventricular rupture, and malignant arrhythmias [4]. Recently, an in-hospital risk score for TTS has been developed from the German and Italian stress cardiomyopathy registry. The authors found three major risk factors (male gender, right ventricular involvement, and history of neurological disorders) and a minor risk factor (LVEF <45%) (Fig. 7) [100]. Some additional prognostic parameters are troponins ten times higher than average values, physical trigger, abnormalities of E/e’ ratio, and mitral insufficiency. It has been noted that TTS induced by physical triggers and neurological issues have a higher long-term mortality rate than emotional triggers compared to acute coronary syndromes [101]. Furthermore, those cases of TTS associated with other critical diseases seem to show a worse short-term prognosis and clinical presentation compared to primary TTS [102].

Cardiogenic shock [7], LVOTO [52], major arrhythmias (ventricular tachycardia or fibrillation) [75], pulmonary edema [103], intraventricular thrombi [104], and free left ventricular wall rupture are common in adverse hospital events [105].

**Arrhythmias**. Major ventricular arrhythmias are predictive of worse outcomes in the short term period; and occur in 3–8.6% of cases and frequently result in death [40]. These events often occur in the subacute phase and are associated with T wave inversion and QTc prolongation. Some believe that TTS is a form of acquired long QTc [106]. Some comparative studies from cardiac MRIs have shown, that the presence of edema is related repolarization inhomogeneity, thus creating the substrate for arrhythmias [107]. A large retrospective study showed that almost 26% of patients would later develop Atrial Fibrillation (AF) (6.9%), Ventricular tachycardia (VT) (3.2%), Atrial flutter (1.9%) and ventricular fibrillation [108]. Data from a multicenter registry has shown that both long- and short-term mortality rate in patients with TTS suffering from arrhythmias is higher than those not presenting with any arrhythmia [109]. The link between AF and TTS is not fully understood; AF might be caused by adrenergic stimulation and electric and structural remodeling of the left atrium. Atrial fibrillation leads to a higher ventricular rate and loss of atrial contribution to ventricular filling, higher neuro-hormonal response, worse mitral insufficiency, and thromboembolic events. The latter is the second most frequent complication of TTS, reaching 2.8% at 30 days and 4.2% at 12 months [104].

**Recurrence and survival rates**. The recurrence rate reaches 5% (3–8%), mostly from 3 months up to 4 years from the first event [110]. The recurrences often involve different areas of the myocardium, suggesting that the presentation is unrelated to varying distributions of adrenergic receptors [111, 112]. The long-term prognosis of these patients seems to be higher than the general population, probably due to the persistence of edema and abnormalities such as those observed on strain imaging [113]. Unfortunately, long-term survival rates are not available. In the Takotsubo registry, the annual mortality rate reaches 5.6% [114]. The death rate for TTC patients is higher than previously thought, and long-term mortality seems to be higher than that of STEMI patients [115]. Long-lasting mild contractile dysfunction, a persistent increase in NT-proBNP, persistent dysfunction of myocardial energetics, and cardiac fibrosis, may all affect long-term quality of life.

**Fig. 8.**
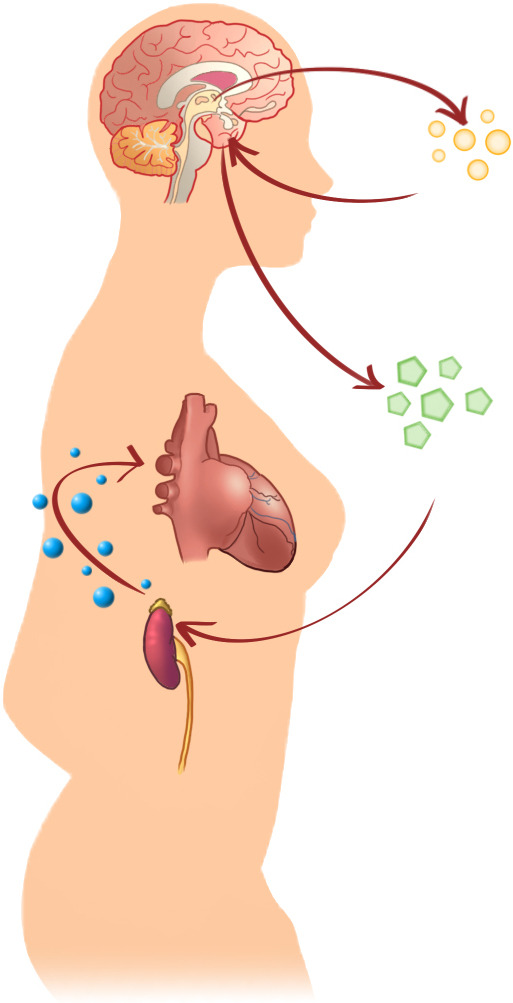
**Heart and brain relationship in Takotsubo**.

**Heart and brain: a close relationship in takotsubo syndrome and the role of PNEI (psychoneuroendocrinoimmunology) **(See Fig. 8).

The link between the heart and brain in TTS has been established since high critical levels of catecholamines have been recognized as a fundamental part of the syndrome. Nevertheless, the same relationship has also been found in a patient with MI and Killip Class III [11]. The substrate on which the psychological stressor acts plays a central role. The thresholds for stress tolerance are part of a complex mechanism not yet completely known, but may be important in some cases [116]. The incidence of Takotsubo syndrome is higher in cohorts of patients with stress and anxiety disorders than in general populations [7]. Individuals predisposed to a higher increase in norepinephrine and epinephrine also have higher levels of resting catecholamines and suffer more frequently from panic disorders [117]. Acute emotional stressors have been shown to induce the activation of different anatomic structures in the central and autonomic nervous systems [78]. The perception of a traumatic event by the cortex triggers the subcortical cerebral circuit through the networks that control the emotions and the functions of the visceral systems, whose activation triggers the release of norepinephrine. Specifically, the cognitive centers of the brain and the hypothalamic-pituitary-adrenal (HPA) axis determine how much epinephrine and norepinephrine are released in response to a given stress. The fundamental anatomic structures involved in interpreting a stimulus as threatening are the neocortex, limbic system, reticular formation, brainstem, and spinal cord [116, 118]. Suzuki *et al*. [119] demonstrated a significant increase in cerebral blood flow in the hippocampus, brainstem, and basal ganglia in the acute phase of Takotsubo. These changes were still present in the chronic phase of the syndrome even after the disappearance of cardiac wall motion abnormalities [119]. The locus coeruleus is the leading site for synthesizing norepinephrine in the brain. It receives afferents from the hypothalamus, cingulate gyrus, and amygdala, allowing emotional stressors to trigger noradrenergic responses, and its activation leads to increased norepinephrine secretion [120]. Another sympathetic neural pathway descends from the posterior hypothalamus through the cranial and sacral spinal cord regions and triggers norepinephrine release. Sympathetic cardiac innervation originates mainly in the right and left stellate ganglia. These fibers travel along the epicardial vascular structures of the heart into the underlying myocardium and end as sympathetic nerve terminals reaching the heart muscle and coronary circulation [121].

A recent PET study revealed an increased stress-related neuronal activity years before disease onset and may constitute a previously overlooked TTS risk factor [122]. Amplifying the sympathetic, neurohormonal, and inflammatory effects of stressors, such as increased upregulation of amygdalar activity, may predispose to TTS [122]. In addition, as stated by Tawakol, increased amygdalar activity independently and effectively predicted cardiovascular disease events in this first longitudinal investigation to associate regional brain activity with eventual cardiovascular disease. A pathway that includes vascular inflammation is one in which amygdalar activity is partially engaged [123]. Using magnetic resonance imaging (MRI) of the brain, Templin *et al*. [124] assessed differences in the brain regions in charge of emotional processing and the integration of the limbic and autonomic systems in patients with a history of TTS compared to a control group. A decline in connections in the limbic system was corroborated by an analysis of structural brain connectivity [124]. Some common abnormalities in the limbic network involving areas such as the insula, amygdala, cingulated cortex, and hippocampus, Campo [125] have been found in patients suffering from TTS and psychiatric and neurological disorders. This is fundamental since those areas are crucial in controlling emotional and cognitive processes and regulating the autonomic system [28]. Additionally, the study of connections in the left amygdala, both hippocampi, left parahippocampal gyrus, left superior temporal pole, and right putamen revealed a decrease in these connections in individuals with TTS [126].

Dichtl *et al*. [127] compared the volumetric differences in gray matter in TTS patients. Compared to controls, TTS patients had significantly less gray matter volume in many regions, especially the right middle frontal gyrus, which included the right insula, left central opercular cortex, right paracingulate gyrus, right and left thalamus, left amygdala, and right subcallosal cortex. These authors recently described modifications in functional connectivity in patients with TTS during the acute stage of the illness. Graph analysis revealed reduced functional connectivity in patients compared to controls, especially in connections from the right anterior IC, temporal lobes, and right precuneus. The researchers found the right insula connected to the sympathetic autonomic tone to have volumetric and functional modifications [127].

It has been demonstrated that TTS patients have fewer changes in pulse pressure in noninvasive tests such as the Valsalva maneuver, static handgrip activities, or tilt tests. This may be significant since they can help evaluate autonomic system function. An inadequate autonomic system response to stress might weaken the heart’s ability to pump blood, which can impede pressure response [128].

To firther underline the strong link between the heart and the brain, 27% [8] of patients suffering from TTS have a neurologic disorder, and 43% [8] have a psychiatric disorder. Moreover, there is a high prevalence of type-D-personality [129], characterized by social inhibition and negative emotions. The correlation between these abnormalities and the syndrome remains unknown, but the relationship between the heart and brain is far more complex. It has been shown that patients suffering from depression have an exaggerated norepinephrine response to emotional stress [130]. Furthermore, TTS often leads to stroke [16], subarachnoid hemorrhage [131] and seizures [132].

In addition, contraction band necrosis, one of the pathological hallmarks of TTS, has also been found in pheochromocytoma [133] and subarachnoid hemorrhage [134], all entities caused by a catecholamine excess [118].

Some miRs (miR16 and miR26a) are up-regulated in stress and depression [135], suggesting the etiologic connection between TTS and neuropsychiatric disorders.

Since there is a psychological component if TTS, some authors have suggested using antidepressant drugs. Since many of these drugs inhibit catecholamine reuptake, they could increase the risk of recurrence [84]. Whether anti-depressants or other psychiatric drugs might provide clinical benefit in patients TTS is controversial. In summary, the Takotsubo syndrome derives from an integrated network of interactions between the individual’s psychology, nervous, and endocrine systems. Through different anatomical structures, the stressor is identified and, depending on the substrate it finds, will cause cardiac damage, also known as the “broken heart syndrome”.

## 6. Conclusions

There are still many open questions to be answered about this syndrome. Among the most important is the risk of recurrence due to an incorrect pharmacological therapy during the acute and chronic phases, the risk of permanent myocardial damage (presence of scar on the MRI), and possible complications (similar to an MI). Fortunately, in most cases, the ejection fraction improves within days or months. However, in some cases, an irreversible impairment of cardiac function is observed. In half of the patients, complications are expected, which have to be detected immediately to prevent a poor prognosis. It is still unclear if some patients may be predisposed to this syndrome, nor do we know the key factor that causes their susceptibility. One of the most critical aspects of TTS is the emotional and psychological involvement that follows the event. Several factors can contribute to generating that sense of fear and dissatisfaction related to the insufficient knowledge of the clinical and psychological implications of TTS. Different elements can enhance this psychological condition: few data are available on this condition, and it is not infrequent that many doctors and nurses underestimate this pathology. There no randomized clinical trials to guide both diagnostic and therapeutic algorithms. Few data are accessible for elaborating a follow-up strategy. Other misunderstandings may derive from inappropriate definitions, like “broken heart syndrome” and under-evaluating the potential complications of this condition, which can be potentially fatal. These patients are “hyper-emotive”, making them feel unsatisfied and they may be considered hypochondriacs. No educational training program has been developed to help these patients minimize their stress reactions.

The damage caused by catecholamines in TTS is still unknown. There are no definitive data about the role of altered molecular mechanisms and cellular death in TTC and their relationship to recovery of cardiac function. Recent TTS neuroimaging research has produced strong evidence for structural and functional changes in the limbic system’s stress-related brain regions. Many issues remain, even though these discoveries have shed light on the potential role of the brain-heart axis in the etiology of TTS. Expanding diagnostic and treatment options may be aided by additional research in this field. Neuroimaging techniques could be used along with current diagnostic methods to accurately identify the mechanisms involved in the acute phase of TTS by detecting circulating microRNAs.
